# Fine Tuning the Energy Barrier of Molecular Nanomagnets *via* Lattice Solvent Molecules

**DOI:** 10.1038/s41598-017-15852-1

**Published:** 2017-11-14

**Authors:** Cai-Ming Liu, De-Qing Zhang, Dao-Ben Zhu

**Affiliations:** Beijing National Laboratory for Molecular Sciences, Center for Molecular Science, Key Laboratory of Organic Solids, Institute of Chemistry, CAS Research/Education Center for Excellence in Molecular Science, Chinese Academy of Sciences, No. 2 1st North Street, Zhongguancun, Beijing 100190 China

## Abstract

Solvents play important roles in our lives, they are also of interest in molecular materials, especially for molecular magnets. The solvatomagnetic effect is generally used for trigger and/or regulation of magnetic properties in molecule-based systems, however, molecular nanomagnets showing solvatomagnetic effects are very difficult to obtain. Here we report four 3d-4f heterometallic cluster complexes containing ROH lattice solvent molecules, [Cu_3_Tb_2_(H_3_L)_2_(OAc)_2_(hfac)_4_]∙2ROH {H_6_L = 1,3-Bis[tris(hydroxymethyl)methylamino]propane, hfac^−^ = hexafluoroacetylacetonate; R = CH_3_, **1**; R = C_2_H_5_, **2**; R = C_3_H_7_, **3**; R = H, **4**}. Single-molecule magnet (SMM) properties of these four complexes were observed to be dependent on the ROH lattice solvent molecule. There is an interesting magneto-structural correlation: the larger the R group, the higher the energy barrier. For the first time, the solvatomagnetic effect is used for the continuous fine adjustment of the energy barrier of 0D molecular nanomagnets. Additionally, [Cu_3_Dy_2_(H_3_L)_2_(OAc)_2_(hfac)_4_]∙2MeOH (**5**), an analogue of [Cu_3_Tb_2_(H_3_L)_2_(OAc)_2_(hfac)_4_]∙2MeOH (**1**), is also reported for comparison.

## Introduction

Solvents, especially water, are critical to the origins of life, and they have penetrated into all aspects of human life. Besides as reaction mediums and extracting agents, chemical solvents are also of interest in molecular materials. For example, in the field of molecular magnets they can be utilized as the terminal ligand to complete the coordination configuration^1–4^; and they can also serve as guest or lattice molecules to adjust magnetic properties^5–9^. The solvatomagnetic effect is very interesting because solvent molecules can be used for trigger and/or regulation of magnetic properties while the molecular magnetic structure is always maintained. Therefore, molecular magnets showing solvatomagnetic effects can be used as molecule devices, molecular switches and/or molecular sensors. Naturally, solvatomagnetic effects are often found in porous metal-organic frameworks (MOFs) in which solvent molecules are guest molecules^5–9^, while low-dimensional systems with solvatomagnetic effects are more difficult to obtain due to the lack of pores. Recently, we found a chain-like azido-bridged manganese(III) coordination polymer showing both solvatomagnetic effect and spin-glass behaviour^10^. In studies of single molecule magnets (SMMs)^11^, we also hope to explore SMM systems with solvatomagnetic effects. However, it is a great challenging task because most SMMs reported are concentrated on zero-dimensional (0 D) cluster or mononuclear systems.

**Figure 1:**
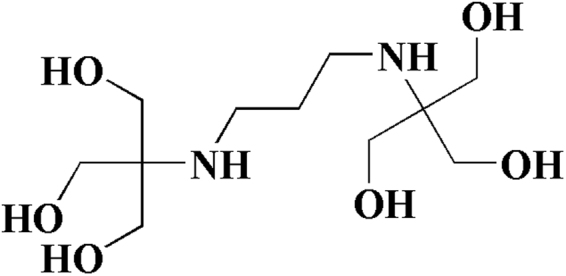
Molecular structure of H_6_L.

It is well known that SMMs are of great potential for technological applications in high-density information storage, quantum computing and spintronics^12–17^; and the energy barrier leading to magnetic bistability and slow magnetic relaxation is a pivotal parameter. Therefore, except enhancing the relaxation energy barrier and increasing the blocking temperature^18–21^, tuning the relaxation energy barrier is another important target in the molecular nanomagnet field^22–26^. Surprisingly, systematic studies of SMMs with the same magnetic structure are still rare, however, some factors such as the electron-withdrawing effect^27^, the electrostatic potential of the key coordination atom^28^ have been observed to be able to modulate SMMs’ energy barriers recently. Regarding structures and magnetic properties may be affected by a small change of circumstance, solvent molecules may also be used to adjust SMMs’ properties. To the best of our knowledge, a direct correlation between energy barriers and different lattice solvent molecules of 0D molecular nanomagnets has never been documented, though a 3D Dy(III) MOF-type SMM was found to show an obvious solvatomagnetic effect in 2015^29^, and guest-dependent single-ion magnet behaviours were observed in a 2D cobalt(II) coordination polymer in 2016^30^. Herein we describe the lattice-solvent effect of ROH molecules (R = CH_3_, **1**; R = C_2_H_5_, **2**; R = C_3_H_7_, **3**; R = H, **4**) on the energy barrier of 0D SMMs with the same magnetic structure [Cu_3_Tb_2_(H_3_L)_2_(OAc)_2_(hfac)_4_] {H_6_L = 1,3-Bis[tris(hydroxymethyl)methylamino]propane, Fig. 1; hfac^−^ = hexafluoroacetylacetonate}. Fine adjustment of the energy barrier (from 25.7 K to 33.1 K, *H*_dc_ = 0 Oe) in this [Cu_3_Tb_2_(H_3_L)_2_(OAc)_2_(hfac)_4_] SMM system was achieved by changing the ROH lattice solvent molecule. A similar [Cu_3_Dy_2_(H_3_L)_2_(OAc)_2_(hfac)_4_] SMM system was also studied, but only the crystal structure of [Cu_3_Dy_2_(H_3_L)_2_(OAc)_2_(hfac)_4_]∙2MeOH (**5**) was successfully solved; complex **5** also exhibits slow magnetic relaxation under zero dc field, with the energy barrier of 30.0 K, a relatively high value for reported relaxation barriers of the Cu-Dy heterometallic SMMs.

## Results and Discussion

### Preparation

Bis-tris propane (H_6_L), an universal ligand due to flexible polydentate coordination sites, has been used to bind not only 3d transition metal ions^31,32^ but also 4 f lanthanide metal ions^33^. Furthermore, it can also be utilized to construct 3d-4f heterometallic complexes^34^. Recently, Murrie *et al*. reported a series of 3d-4f complexes formulated as {Ln_2_Cu_3_(H_3_L)_2_X_n_} (X = OAc^−^, Ln = Gd, Tb or X = NO_3_^−^, Ln = Gd, Tb, Dy, Ho, Er)^35^; they found that changing the auxiliary ligand OAc^−^ through NO_3_^−^ may lead to a remarkable improvement of the energy barrier of {Tb_2_Cu_3_(H_3_L)_2_X_n_} (X = OAc^−^ and NO_3_^−^) complexes, which suggests that the anion co-ligand has a great impact on the energy barrier of {Tb_2_Cu_3_(H_3_L)_2_X_n_} SMMs. In the recent process of pursuing new SMMs, we observed that using Ln(OAc)(hfac)_2_(H_2_O)_2_ as the lanthanide (III) salt source may lead mixed co-ligands OAc^−^ and hfac^−^ into 3d-4f heterometallic clusters effectively^36^. Therefore, we adopted this synthesis strategy to obtain the [Cu_3_Tb_2_(H_3_L)_2_(OAc)_2_(hfac)_4_] SMM with different ROH lattice solvent molecules (methanol, ethanol and isopropyl alcohol), in which not only the OAc^−^ anion but also the hfac^−^ anion are co-ligands. Notably, our synthetic procedures were completed at room temperature rather than at 60 °C used by Murrie group^35^. Products using methanol, ethanol and isopropyl alcohol as reaction solvents were [Cu_3_Tb_2_(H_3_L)_2_(OAc)_2_(hfac)_4_]∙2MeOH (**1**), [Cu_3_Tb_2_(H_3_L)_2_(OAc)_2_(hfac)_4_]∙2EtOH (**2**) and [Cu_3_Tb_2_(H_3_L)_2_(OAc)_2_(hfac)_4_]∙2iso-C_3_H_7_OH (**3**), respectively; while [Cu_3_Tb_2_(H_3_L)_2_(OAc)_2_(hfac)_4_]∙2H_2_O (**4**) was quantitatively transformed from complex **1** by taking place of methanol molecules with water molecules. In order to yield [Cu_3_Tb_2_(H_3_L)_2_(OAc)_2_(hfac)_4_] SMMs with larger ROH lattice solvent molecules, other ROH solvents such as isobutyl alcohol, n-butyl alcohol and isoamyl alcohol were also used instead of methanol for **1**, but no any crystalline products could be obtained. Furthermore, the [Cu_3_Dy_2_(H_3_L)_2_(OAc)_2_(hfac)_4_] SMM system was also explored, but only the crystal structure of [Cu_3_Dy_2_(H_3_L)_2_(OAc)_2_(hfac)_4_]∙2MeOH (**5**) was successfully solved, the crystal structure of [Cu_3_Dy_2_(H_3_L)_2_(OAc)_2_(hfac)_4_] SMMs with other lattice solvent molecules (H_2_O, ethanol and isopropyl alcohol) could not be obtained due to the severe twinning phenomenon.

### Structural description

**Figure 2:**
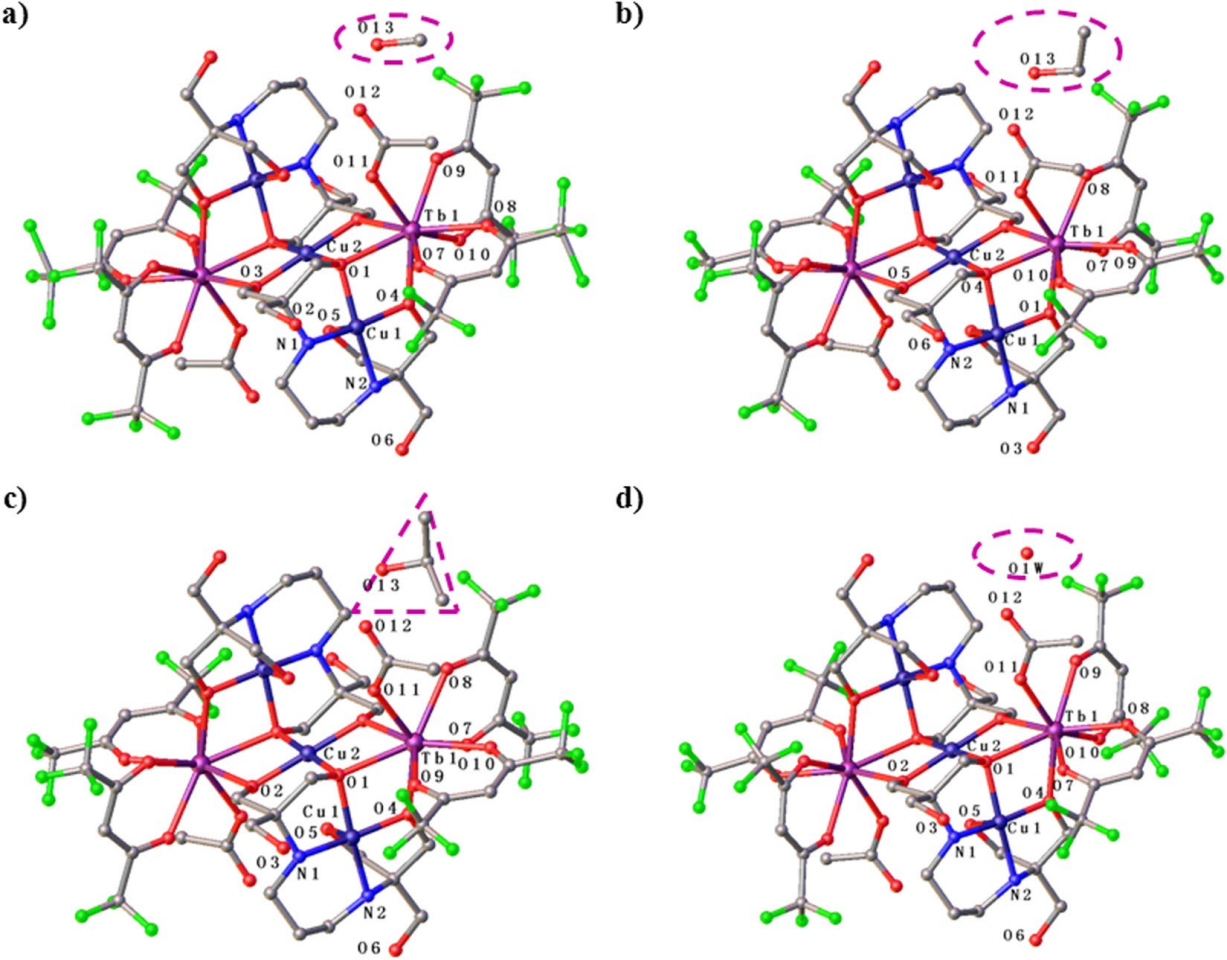
Crystal structures of **1** (**a**), **2** (**b**), **3** (**c**) and **4** (**d**). All lattice solvent molecules are highlighted, and all H atoms are omitted for clarity.

All [Cu_3_Tb_2_(H_3_L)_2_(OAc)_2_(hfac)_4_]∙2ROH SMMs have the main structure [Cu_3_Tb_2_(H_3_L)_2_(OAc)_2_(hfac)_4_] (Fig. 2). Therefore, the structure of [Cu_3_Tb_2_(H_3_L)_2_(OAc)_2_(hfac)_4_]∙2MeOH (**1**) is chose to be described in detail. In the main structure [Cu_3_Tb_2_(H_3_L)_2_(OAc)_2_(hfac)_4_], a {Cu_3_(H_3_L)_2_} linear unit is formed through bridging two terminal {Cu(H_3_L)}^−^ fragments using a central Cu^2+^ ion, then two Tb^3+^ ions link to this {Cu_3_(H_3_L)_2_} linear unit in the opposite direction, in which each Tb^3+^ ion connects with the central Cu^2+^ ion and one external Cu^2+^ ion through sharing one *μ*_3_-O atom and one *μ*-O atom from one H_3_L^3−^ ligand, and one *μ*_3_-O atom from the other H_3_L^3−^ ligand (Fig. 2a), similar to those in {Ln_2_Cu_3_(H_3_L)_2_X_n_}^35^. The eight-coordinate sphere of each Tb^3+^ ion is finally completed by two hfac^−^ anions and one OAc^−^ anion. Shape software^37^ was adopted to calculate the Tb(III) coordination polyhedron, giving a triangular dodecahedron as the most likely configuration for complex **1**, and the deviation value from the ideal *D*_2d_ symmetry is 1.015 (Table S1, SI). It is worth noting that the Tb(III) coordination polyhedron can also be viewed as a biaugmented trigonal prism, but with the deviation value of 1.756 from the ideal *D*_2d_ symmetry. Moreover, the calculation result for the Tb(III) coordination polyhedra of complexes **2**–**4** using Shape software^37^ are listed in Tables S2–S4 (SI), respectively.

The external Cu atom, in a distorted square-pyramidal configuration, is coordinated with two N atoms and two *μ*-O atoms from one H_3_L^3−^ ligand, forming the base of the pyramid; whereas the third *μ*-O atom from the same H_3_L^3−^ ligand occupying the apical site. The central Cu^2+^ ion is coordinated by two *μ*_3_-O atoms and four *μ*-O atoms from two H_3_L^3−^ ligands, generating a distorted octahedral geometry, in which two *μ*-O atoms bridging the central Cu atom and the external Cu atom are in the Jahn-Teller axis’ direction, with the long Cu-O bond distance of 2.665 Å for complex **1**.

There are hydrogen bonds between the methanol O atom and the carboxylate O atom with the O_methanol_…O_carboxylate_ distance of 2.803 Å and between the methanol O atom and the N atom from the H_3_L^3−^ ligand with the O_methanol_…N distance of 2.937 Å for complex **1**. Similar hydrogen bonds were observed between the ethanol O atom and the carboxylate O atom with the O_ethanol_…O_carboxylate_ distance of 2.788 Å and between the ethanol O atom and the N atom from the H_3_L^3−^ ligand with the O_ethanol_…N distance of 2.941 Å for **2**; between the isopropyl alcohol O atom and the carboxylate O atom with the O_isopropyl alcohol_…O_carboxylate_ distance of 2.817 Å and between the isopropyl alcohol O atom and the N atom from the H_3_L^3−^ ligand with the O_isopropyl alcohol_…N distance of 2.922 Å for **3**; and between the water O atom and the carboxylate O atom with the O_water_…O_carboxylate_ distance of 2.866 Å and between the water O atom and the N atom from the H_3_L^3−^ ligand with the O_water_…N distance of 2.944 Å for **4**. These weak intermolecular interactions play important roles in not only stabilization of crystal structures but also adjustment of magnetic properties for complexes **1**–**4**.

Complex **5** has the same structure as **1**, but Dy instead of Tb is used (Fig. S1, SI). The Dy-O bond distance (average 2.357 Å) in **5** is slightly smaller than the Tb-O bond length (average 2.368 Å) in **1** owing to the lanthanide contraction effect. The Dy(III) coordination polyhedron can also be described as a triangular dodecahedron with the deviation value of 0.975 from the ideal *D*_2d_ symmetry (Table S5, SI). This value is a little smaller than that of **1** (1.015), indicating that the Dy(III) coordination polyhedron in **5** is closer to a triangular dodecahedron than the Tb(III) coordination polyhedron in **1**. The deviation value from the ideal *D*_2d_ symmetry for a biaugmented trigonal prism is 1.735 for **5**, also a little smaller than that of **1** (1.756). Similar to **1**, there are also hydrogen bonds between the methanol O atom and the carboxylate O atom with the O_methanol_…O_carboxylate_ distance of 2.796 Å and between the methanol O atom and the N atom from the H_3_L^3−^ ligand with the O_methanol_…N distance of 2.937 Å for complex **5**.

### Magnetic properties

**Figure 3:**
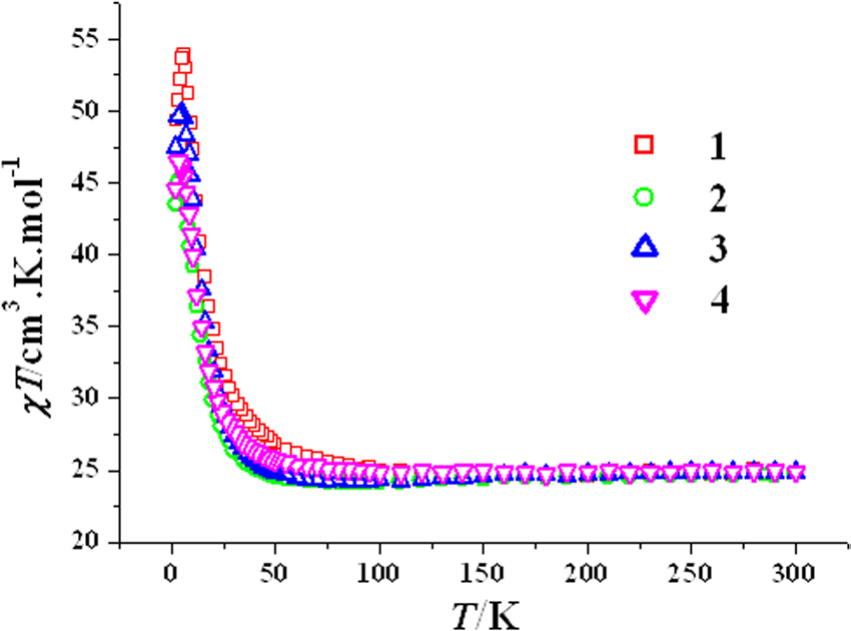
Plot of *χT* vs *T* for **1–4**.

The direct current (dc) variable-temperature magnetic susceptibility of complexes **1**–**4** was measured at 1000 Oe applied field (Fig. 3). The room temperature *χT* values of the complexes **1** (24.91 cm^3^ K mol^−1^), **2** (24.85 cm^3^ K mol^−1^), **3** (24.84 cm^3^ K mol^−1^) and **4** (24.90 cm^3^ K mol^−1^) are slightly larger than the theoretical value of 24.77 cm^3^ K mol^−1^ for three noninteracting Cu^2+^ ions (*g* = 2.0) and two uncoupled Tb^3+^ ions (^7^ *F*_6_, *J* = 6, *L* = 3, *S* = 3, *g* = 3/2). As shown in Fig. 3, upon cooling, the *χT* product almost keeps a constant value or just slightly lowers; however, below about 50 K, a rapid rise appears until reaches the maximum values of 53.92 cm^3^ K mol^−1^ at 6.0 K for **1**, 45.14 cm^3^ K mol^−1^ at 4.0 K for **2** and 49.80 cm^3^ K mol^−1^ at 4.0 K for **3**; the *χT* values then decline to 49.34 cm^3^ K mol^−1^ at 2.0 K for **1**, 43.48 cm^3^ K mol^−1^ at 2.0 K for **2** and 47.48 cm^3^ K mol^−1^ at 2.0 K for **3**. Exceptionally, complex **4** does not reach the maximum value until 2.0 K (44.60 cm^3^ K mol^−1^). These magnetic behaviours are very similar to those of {Tb_2_Cu_3_(H_3_L)_2_X_n_} (X = OAc^−^ and NO_3_^−^)^35^, suggesting that all four complexes are also ferromagnetic. The small difference in dc magnetic susceptibilities of **1**–**4** means that there is a solvatomagnetic effect in this [Cu_3_Tb_2_(H_3_L)_2_(OAc)_2_(hfac)_4_] SMM system.

**Figure 4:**
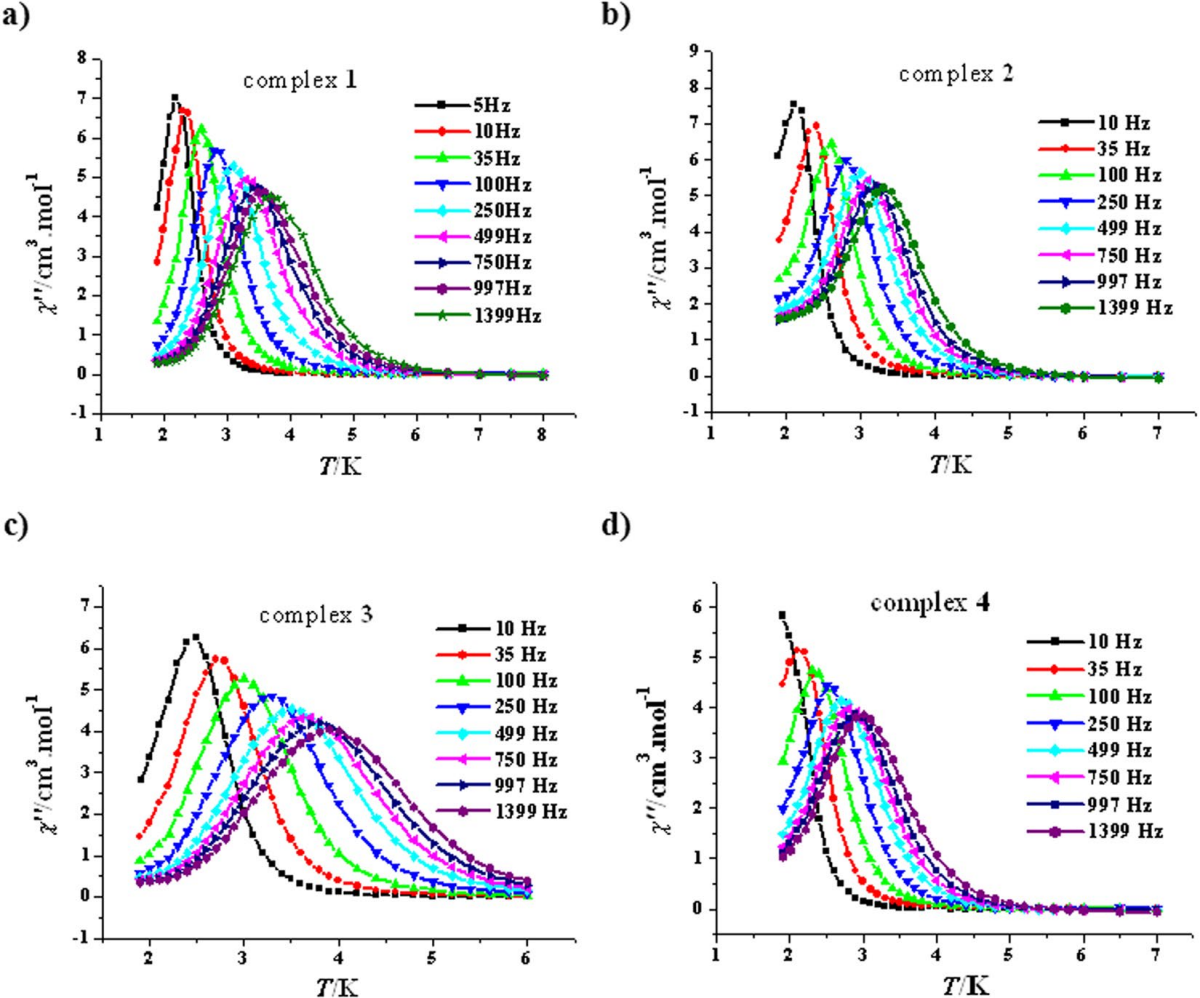
Plots of *χ*′′ vs *T* for **1** (**a**), **2** (**b**), **3** (**c**) and **4** (**d**) (*H*_dc_ = 0 Oe, *H*_ac_ = 2.5 Oe).

**Figure 5:**
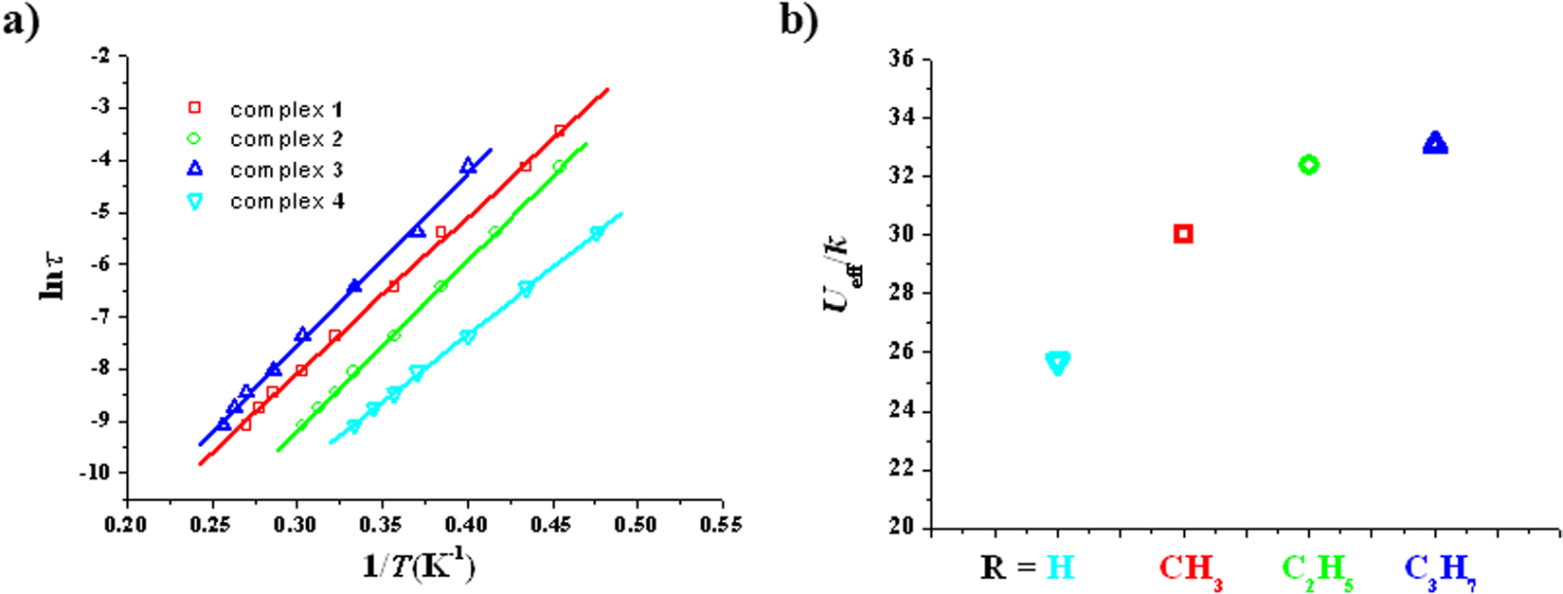
Plot of ln(*τ*) vs 1*/T* for **1–4** (**a**), the solid lines represent the best fitting with the Arrhénius law; magneto-structural correlation between *U*_eff_/k values and the R groups of ROH solvent molecules (**b**).

The solvatomagnetic effect could also be detected by alternating current (ac) magnetic susceptibility investigations. Both the in-phase (*χ′*, Fig. S2, SI) and the out-of-phase (*χ′′*, Fig. 4) of variable-temperature ac magnetic susceptibility for **1**–**4** are frequency-dependent in zero dc field, indicating slow magnetic relaxation typical for SMMs. Such thermally induced relaxation was fitted with the Arrhenius law, *τ* = *τ*_0_exp(*U*_eff_/*kT*), extracting *U*_eff_/*k* values of 30.0(0.4) K for **1**, 32.4(0.2) K for **2**, 33.1(0.7) K for **3** and 25.7(0.2) K for **4** as well as *τ*_0_ values of 3.7(0.2) × 10^−8^ s for **1**, 6.2(0.1) × 10^−9^ s for **2**, 2.6(0.3) × 10^−8^ s for **3** and 2.3(0.1) × 10^−8^ s for **4** (Fig. 5a). All four *τ*_0_ values are within the normal range for SMMs/SIMs (10^−5^–10^−11^ s)^13^. A comparison of the effective barrier value for complexes **1**–**4** with the R group of the ROH lattice solvent molecules (R = H, **4**; R = CH_3_, **1**; R = C_2_H_5_, **2** and R = C_3_H_7_, **3**) reveals an important magneto-structural correlation for this [Cu_3_Tb_2_(H_3_L)_2_(OAc)_2_(hfac)_4_] SMM system: The larger the R group in ROH, the higher the energy barrier of the [Cu_3_Tb_2_(H_3_L)_2_(OAc)_2_(hfac)_4_]∙2ROH SMM (Fig. 5b). It is noteworthy that either the *U*_eff_/*k* value of **2** or the *U*_eff_/*k* value of **3** is one of the largest values so far for the Cu-Tb heterometallic SMMs in zero dc field, just smaller than that of (NMe_4_)_2_[Tb_2_Cu_3_(H_3_L)_2_(NO_3_)_7_(CH_3_OH)_2_](NO_3_) (36 K)^35^; the *U*_eff_/*k* value of **1** is also remarkable, which is comparable with that of [Cu_2_(valpn)_2_Tb_2_(N_3_)_6_]·2CH_3_OH [H_2_valpn = 1,3-propanediylbis(2-iminomethylene-6-methoxyphenol)] (30.1 K, *H*_*dc*_ = 0 Oe)^38^. In many cases^39–44^, a dc field is necessary for 3d-4f heterometallic complexes to display magnetic relaxation because of the obvious quantum-tunnelling effects.

Simplified theoretical investigations by Murrie group suggested that the magnetic bistability in the [Cu_3_Tb_2_(H_3_L)_2_X_n_] system is not because of single-ion behaviours, and both the Cu···Cu and Cu···Tb ferromagnetic interactions maybe quench the tunnel splitting, which are similar to acting as an internal applied field, inducing to zero-field SMM behaviours^35^. Nevertheless, the difference of the Tb^3+^ coordination configurations has influence on the SMM characteristics^35^. Owing to great difficulty for theoretical calculation and comparison of the Cu···Cu and Cu···Tb ferromagnetic couplings^35^, we tried to make a magneto-structural correlation for complexes **1**–**4** using the deviation value from the ideal *D*_2d_ symmetry of the biaugmented trigonal prism for the Tb^3+^ ion and the intermolecular distance as two main structural parameters. As shown in Table 1, the coordination configuration of the Tb^3+^ ions is closer to the biaugmented trigonal prism from **1** to **3**, the corresponding energy barrier value becomes larger from **1** to **3**, indicating the biaugmented trigonal prism configuration in the [Cu_3_Tb_2_(H_3_L)_2_(OAc)_2_(hfac)_4_] SMM system is the dominant configuration; but **4** is a bit unusual, its deviation value (1.735) is comparable with that of **1** (1.756), which suggests that other structural factors such as intermolecular distances need to be considered; as shown in Table 1, the longer the intermolecular distance (defined by the shortest Cu_central_…Cu_central_ separation), the higher the energy barrier; which is in line with the magneto-structural correlation using the R group itself, because larger ROH lattice solvent molecules may enhance intermolecular distances correspondingly.

**Table 1:** Magneto-structural correlation of *U*_eff_/*k* values with two structural parameters.

complex	ROH solvent molecule	the deviation value of biaugmented trigonal prism for the Tb^3+^ ion	the shortest Cu_central_…Cu_central_ separation	*U*_eff_/*k* (K)	*τ*_0_ (s)
**4**	R = H	1.735	10.042	25.7	2.3 × 10^−8^
**1**	R = CH_3_	1.756	10.086	30.0	3.7 × 10^−8^
**2**	R = C_2_H_5_	1.584	10.244	32.4	6.2 × 10^−9^
**3**	R = C_3_H_7_	1.496	10.309	33.1	2.6 × 10^−8^

**Figure 6:**
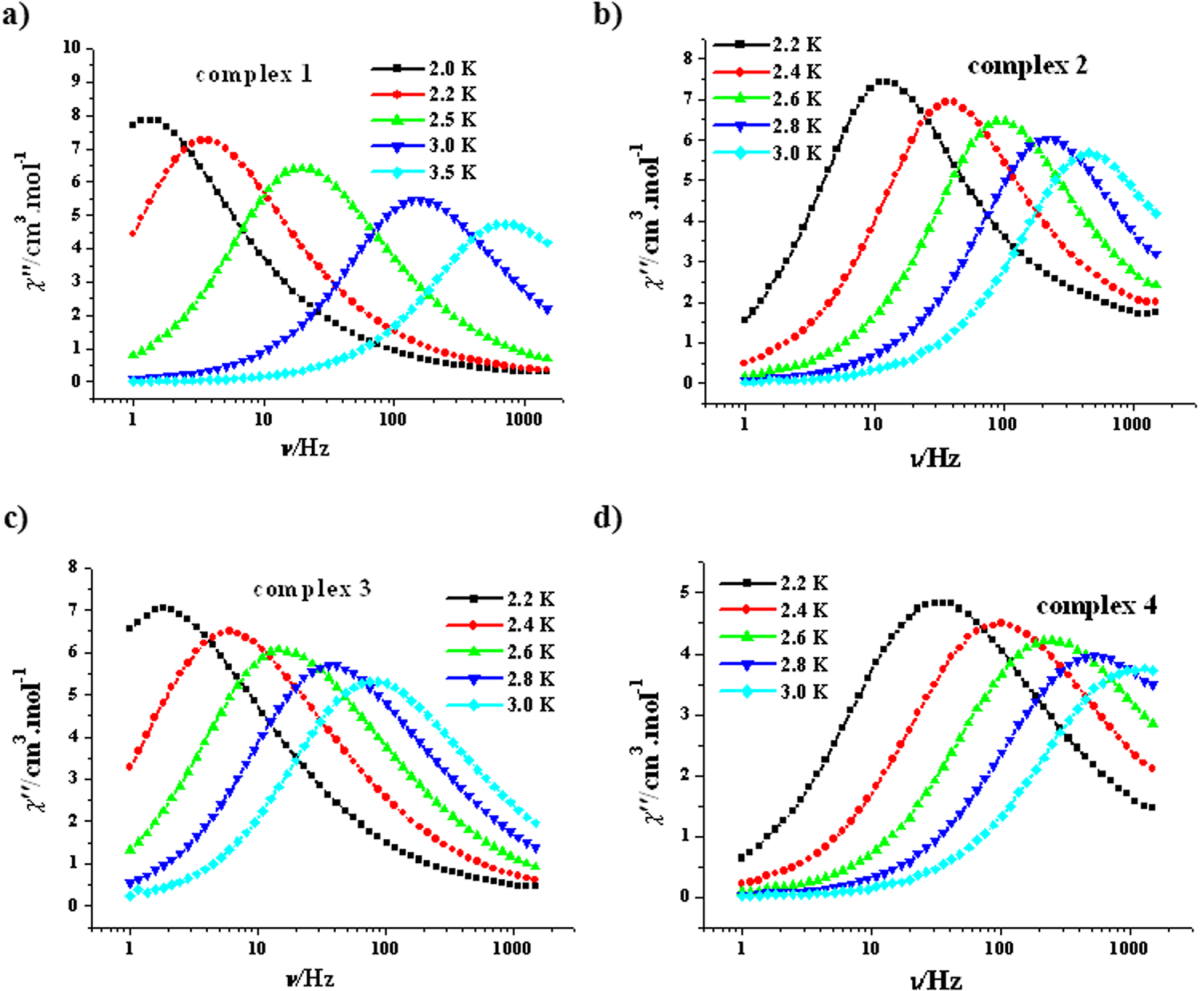
Plots of *χ*′′ vs *ν* for **1** (**a**), **2** (**b**), **3** (**c**) and **4** (**d**) (*H*_dc_ = 0 Oe, *H*_ac_ = 2.5 Oe).

The SMM properties of **1–4** were also evaluated by the parameter *Φ* = (Δ*T*_f_/*T*_f_)/Δ(log*f*)^45^, where *f* represents the frequency and *T*_f_ the peak temperature of *χ*″ curve; the *Φ* values of **1, 2, 3** and **4** are 0.18, 0.17, 0.17 and 0.21, respectively, which support the superparamagnet behaviour of these SMMs (*Φ* > 0.1), but exclude any spin glass properties (*Φ* ≈ 0.01)^45^. Further determinations of ac magnetic susceptibility revealed that the variable-frequency *χ*″ signals of **1**–**4** are evidently temperature-dependent (Fig. 6), confirming slow magnetic relaxation of SMMs. The *χ*″ vs *χ*′ plots show classical half-circular curves for all four complexes, indicating a single magnetic relaxation process (Fig. S3, SI). These Cole-Cole plots could be fitted with a generalized Debye model^46,47^. The *α* values are smaller than 0.07 for **2**–**4**, suggesting a single relaxation mechanism; while the *α* values for **1** are from 0.10 to 0.22, indicating a relatively narrow distribution of the relaxation time. In addition, no any hysteresis was observed in the *M* vs *H* plot at 1.9 K for **1**–**4** (Fig. S4, SI).

**Figure 7:**
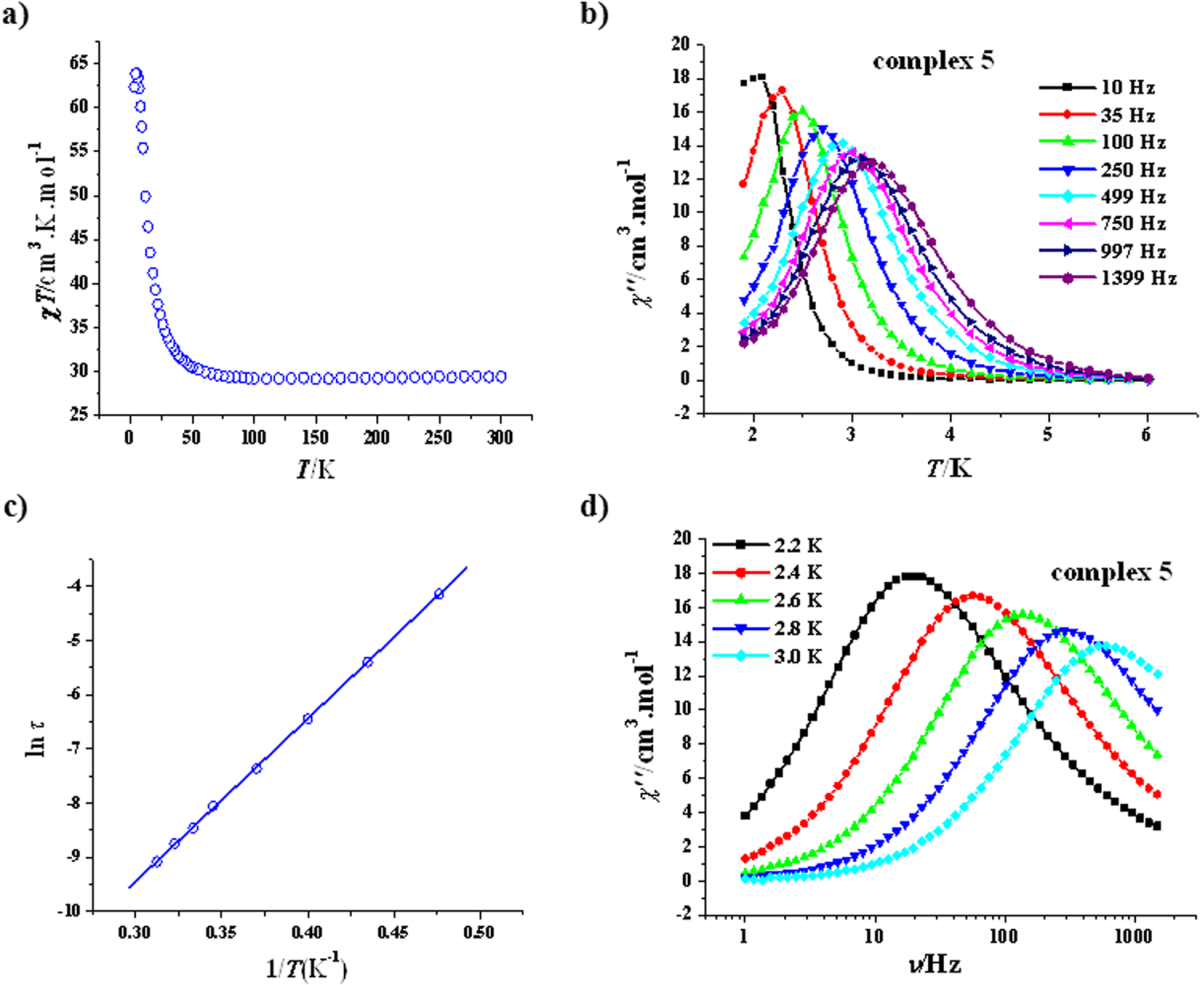
Plot of *χT* vs *T* of **5** (**a**); plot of *χ*′′ vs *T* for **5** (**b**) (*H*_dc_ = 0 Oe, *H*_ac_ = 2.5 Oe); plot of ln(*τ*) vs 1*/T* for **5** (**c**), the solid line represents the best fitting with the Arrhénius law; plot of *χ*′′ vs *ν* for **5** (**d**) (*H*_dc_ = 0 Oe, *H*_ac_ = 2.5 Oe).

The *χT* value at room temperature for complex **5** is 29.44 cm^3^ K mol^−1^ (Fig. 7a), which is in good agreement with the expected value of 29.47 cm^3^ K mol^−1^ for three uncoupled Cu^2+^ ions (*g* = 2.0) and two isolated Dy^3+^ ions (^6^ *H*_15/2_, *J* = 15/2, *S* = 5/2, *L* = 5, *g* = 4/3). When temperature is decreased, the *χT* product decreases very slowly until 110 K (29.20 cm^3^ K mol^−1^), then increases very gently until about 50 K. Below this temperature, the *χT* value rises rapidly, reaching the maximum of 63.92 cm^3^ K mol^−1^ at 5 K and then dropping down to 62.38 cm^3^ K mol^−1^ at 2 K, these magnetic behaviors are similar to those for **1** and (NMe_4_)_2_{Dy_2_Cu_3_(NO_3_)_7_(CH_3_OH)_2_](NO_3_)^35^, and the ferromagnetic coupling obviously exists between the Cu^2+^ ion and the Dy^3+^ ion as well as among the Cu^2+^ ions, similar to that observed in [Gd_2_Cu_3_(H_3_L)_2_(CH_3_COO)_6_]·THF·3H_2_O by Murrie group^35^.

The magnetization dynamics of compound **5** are similar to those of complexes **1**–**4**. Under zero dc field, the appearance of frequency-dependent *χ*′ (Fig. S5, SI) and *χ*″ signals (Fig. 7b) indicates SMM behaviors of **5**. The SMM parameters extracted from the Arrhenius law for **5** are *U*_eff_/*k* = 30.0(0.2) K and *τ*_0_ = 9.7(0.1) × 10^−9^ s (Fig. 7c). The energy barrier value of **5** is comparable with that of **1**, but obviously larger than that of (NMe_4_)_2_{Dy_2_Cu_3_(H_3_L)_2_(NO_3_)_7_(CH_3_OH)_2_](NO_3_) [23.9(0.1) K], whose *χ*″ signals even do not appear peaks in zero dc field^35^. Notably, this *U*_eff_/*k* value is the third high value for the Cu-Dy heterometallic SMMs, after 47 K of [{Dy(hfac)_3_}_2_{Cu(dpk)_2_}] (dpk^−^ = di-2-pyridyl ketoximate)^48^ and 41.6 K of [Cu_4_Dy_4_(vanox)_6_(Hvanox)_2_(NO_3_)_4_(*μ*-HOMe)_2_]·6MeOH (H_2_vanox = 3-methoxy-2-hydroxybenzaldoxime)^49^. Furthermore, this *U*_eff_/*k* value is remarkable larger than those of the Cu-Dy heterometallic SMMs with higher nucleus (<20 K)^50,51^. Additionally, the parameter *Φ* value of 0.16 for **5** supports the SMM nature too.

The variable-frequency ac magnetic susceptibility study of **5** revealed that the *χ*″ signals of **5** are temperature-dependent (Fig. 7d), confirming the SMM behavior of **5**. The Cole-Cole plots were fitted to a generalized Debye model (Fig. S6, SI)^46,47^, giving the *α* values of 0.01–0.08 for **5**, suggesting the magnetic relaxation happens *via* a single relaxation process. Additionally, the *M* vs *H* plot of **5** shows no any hysteresis at 1.9 K (Fig. S7, SI).

## Conclusions

In summary, a mixed OAc^−^/hfac^−^ co-ligands’ synthesis strategy was adopted to prepare 3d-4f heterometallic SMMs based on the 1,3-Bis[tris(hydroxymethyl)methylamino]propane ligand (H_6_L). The ROH lattice solvent molecules (R = H, CH_3_, C_2_H_5_ and C_3_H_7_) in the [Cu_3_Tb_2_(H_3_L)_2_(OAc)_2_(hfac)_4_] SMM system have great influences on the energy barrier; the larger the R group, the higher the energy barrier. We predict that the larger ROH molecule may enlarge the intermolecular distance and can help to change the coordination configuration of the Ln(III) ions through the hydrogen bonding interaction between the ROH lattice solvent molecule and the [Cu_3_Tb_2_(H_3_L)_2_(OAc)_2_(hfac)_4_] main-structural molecule. Our work demonstrates that solvatomagnetic effects can be used to continuously fine-tune energy barriers in SMMs. The discovery is bound to have significances in enhancing and turning energy barriers of molecular nanomagnets *via* chemical methods such as using lattice-solvent effects.

## Methods

### Physical measurements

The elemental analyses were measured on a Vario ELIII elemental analyser. The magnetic susceptibility measurements were carried out on a Quantum Design MPMS-XL5 SQUID magnetometer, and diamagnetic corrections were calculated from Pascal’s constants of all components.

### Synthesis of [Cu_3_Tb_2_(H_3_L)_2_(OAc)_2_(hfac)_4_]∙2MeOH (1)

To a mixture of H_6_L (0.25 mmol) and Cu(ClO_4_)_2_·6H_2_O (0.375 mmol) in 20 mL of MeOH, was added Tb(OAc)(hfac)_2_(H_2_O)_2_ (0.15 mmol), a blue solution was formed after being stirred for 10 min; Et_3_N (0.75 mmol) was then added dropwise, the resultant solution was stirred for 3 h at room temperature and turned violet. Violet plate-like X-ray quality crystals were obtained through slow evaporation of the filtrate at room temperature over 1 week. Yield (25%). Anal. Calcd (%) for C_48_H_64_Cu_3_F_24_N_4_O_26_Tb_2_ (**1**) C 27.75; H 3.11; N 2.70. Found: C 27.80; H 3.14; N 2.67.

### Synthesis of [Cu_3_Tb_2_(H_3_L)_2_(OAc)_2_(hfac)_4_]∙2EtOH (2)

The same synthetic procedure for complex **1** was followed, but using ethanol instead of methanol. Violet plate-like X-ray quality crystals were obtained through slow evaporation of the filtrate at room temperature over 10 days. Yield (27%). Anal. Calcd (%) for C_50_H_68_Cu_3_F_24_N_4_O_26_Tb_2_ (**2**): C 28.52; H 3.26; N 2.66. Found: C 28.55; H 3.29; N 2.63.

### Synthesis of [Cu_3_Tb_2_(H_3_L)_2_(OAc)_2_(hfac)_4_]∙2iso-C_3_H_7_OH (3)

The same synthetic procedure for complex **1** was followed, but using isopropyl alcohol instead of methanol. Violet plate-like X-ray quality crystals were obtained through slow evaporation of the filtrate at room temperature over 15 days. Yield (22%). Anal. Calcd (%) for C_52_H_72_Cu_3_F_24_N_4_O_26_Tb_2_ (**3**): C 29.27; H 3.40; N 2.63. Found: C 29.23; H 3.43; N 2.60.

### Synthesis of [Cu_3_Tb_2_(H_3_L)_2_(OAc)_2_(hfac)_4_]∙2H_2_O (4)

Complex **1** was kept at 60 °C for 6 h, and then exposed on air for 24 h. Violet plate-like X-ray quality crystals of **4** were obtained quantitatively. Anal. Calcd (%) for C_46_H_60_Cu_3_F_24_N_4_O_26_Tb_2_ (**4**): C 26.96; H 2.95; N 2.73. Found: C 27.02; H 2.99; N 2.69.

### Synthesis of [Cu_3_Dy_2_(H_3_L)_2_(OAc)_2_(hfac)_4_]∙2MeOH (5)

The same synthetic procedure for complex **1** was followed, but using Dy(OAc)(hfac)_2_(H_2_O)_2_ instead of Tb(OAc)(hfac)_2_(H_2_O)_2_. Violet plate-like X-ray quality crystals were obtained through slow evaporation of the filtrate at room temperature over 1 week. Yield (28%). Anal. Calcd (%) for C_48_H_64_Cu_3_Dy_2_F_24_N_4_O_26_ (**5**): C 27.66; H 3.09; N 2.69. Found: C 27.69; H 3.11; N 2.67.

### X-ray crystallography

A single crystal with dimensions 0.261 × 0.093 × 0.025 mm^3^ of **1**, 0.178 × 0.063 × 0.024 mm^3^ of **2**, 0.183 × 0.125 × 0.031 mm^3^ of **3**, 0.108 × 0.067 × 0.025 mm^3^ of **4**, and 0.134 × 0.125 × 0.027 mm^3^ of **5** was picked out to mount on a Bruker SMART APEX-CCD diffractometer with Mo-K_*α*_ radiation (*λ* = 0.71073 Å) for data collection at 173(2) K. Empirical absorption corrections from *φ* and *ω* scan were applied. Cell parameters were calculated by the global refinement of the positions of all collected reflections for five complexes. The structures were solved by direct methods and refined by a full matrix least-squares technique based on F^2^ using with the SHELX-2014 program package. All hydrogen atoms were set in calculated positions and refined as riding atoms, and all non-hydrogen atoms were refined anisotropically. CCDC 1574978–1574982 contain the supplementary crystallographic data, which can be obtained free of charge from the Cambridge Crystallographic Data Centre via www.ccdc.cam.ac.uk/data_request/cif.

Crystal data for **1**: *P*−1, *a* = 10.086(2) Å, *b* = 12.463(3) Å, *c* = 15.594(3) Å, *α* = 104.67(3)°, *β* = 94.07(3)°, *γ* = 108.97(3)°, *V* = 1767.9(7) Å^3^, *M*_r_ = 2077.49, *D*_c_ = 1.951 g cm^−3^, *Z* = 1, *R*_1_ = 0.0366 (*I* > 2*σ*(*I*)), *wR*_2_ = 0.0878 (*I* > 2*σ*(*I*)), *S* = 1.080.

Crystal data for **2**: *P*−1, *a* = 10.243(2) Å, *b* = 12.469(3) Å, *c* = 15.602(3) Å, *α* = 101.71(3)°, *β* = 96.50(3)°, *γ* = 110.00(3)°, *V* = 1797.2(6) Å^3^, *M*_r_ = 2105.54, *D*_c_ = 1.945 g cm^−3^, *Z* = 1, *R*_1_ = 0.0498 (*I* > 2*σ*(*I*)), *wR*_2_ = 0.1085 (*I* > 2*σ*(*I*)), *S* = 1.122.

Crystal data for **3**: *P*−1, *a* = 10.309(2) Å, *b* = 12.473(3) Å, *c* = 15.677(3) Å, *α* = 101.80(3)°, *β* = 96.96(3)°, *γ* = 110.19(3)°, *V* = 1811.4(7) Å^3^, *M*_r_ = 2133.58, *D*_c_ = 1.956 g cm^−3^, *Z* = 1, *R*_1_ = 0.0345 (*I* > 2*σ*(*I*)), *wR*_2_ = 0.0797 (*I* > 2*σ*(*I*)), *S* = 1.084.

Crystal data for **4**: *P*−1, *a* = 10.042(2) Å, *b* = 12.480(3) Å, *c* = 15.819(3) Å, *α* = 107.08(3)°, *β* = 99.23(3)°, *γ* = 109.83(3)°, *V* = 1706.2(7) Å^3^, *M*_r_ = 2049.44, *D*_c_ = 1.995 g cm^−3^, *Z* = 1, *R*_1_ = 0.0488 (*I* > 2*σ*(*I*)), *wR*_2_ = 0.0945 (*I* > 2*σ*(*I*)), *S* = 1.153.

Crystal data for **5**: *P*−1, *a* = 10.085(2) Å, *b* = 12.427(3) Å, *c* = 15.581(3) Å, *α* = 104.59(3)°, *β* = 94.21(3)°, *γ* = 108.86(3)°, *V* = 1762.5(7) Å^3^, *M*_r_ = 2084.65, *D*_c_ = 1.964 g cm^−3^, *Z* = 1, *R*_1_ = 0.0321 (*I* > 2*σ*(*I*)), *wR*_2_ = 0.0762 (*I* > 2*σ*(*I*)), *S* = 1.074.

## Electronic supplementary material

Supplementary Information
