# Supplementary material for: Fine Tuning the Energy Barrier of Molecular Nanomagnets via Lattice Solvent Molecules
Source: Sci Rep. 2017 Nov 14;7:15483. doi: 10.1038/s41598-017-15852-1 (PMC5686112; doi:10.1038/s41598-017-15852-1)
Supplement: Supplementary file 1 — Supplementary Information [file 41598_2017_15852_MOESM1_ESM.pdf]

Fine Tuning the Energy Barrier of Molecular Nanomagnets *via* Lattice Solvent Molecules

Cai-Ming Liu,* De-Qing Zhang & Dao-Ben Zhu

Beijing National Laboratory for Molecular Sciences, Center for Molecular Science, Key Laboratory of Organic Solids, Institute of Chemistry, CAS Research/Education Center for Excellence in Molecular Science, Chinese Academy of Sciences, No.2 1st North Street, Zhongguancun, Beijing 100190, China.

Table S1. Continuous Shape Measures' calculation for the Tb(III) ion in complex 1.

Tb structures

OP-8	1 D8h	Octagon
HPY-8	2 C7v	Heptagonal pyramid
HBPY-8	3 D6h	Hexagonal bipyramid
CU-8	4 Oh	Cube
SAPR-8	5 D4d	Square antiprism
TDD-8	6 D2d	Triangular dodecahedron
JGBF-8	7 D2d	Johnson gyrobifastigium J26
JETBPY-8	8 D3h	Johnson elongated triangular bipyramid J14
JBTPR-8	9 C2v	Biaugmented trigonal prism J50
BTPR-8	10 C2v	Biaugmented trigonal prism
JSD-8	11 D2d	Snub diphenoid J84
TT-8	12 Td	Triakis tetrahedron
ETBPY-8	13 D3h	Elongated trigonal bipyramid

Structure [ML8]	OP-8	HPY-8	HBPY-8	CU-8	SAPR-8	TDD-8	JGBF-8	JETBPY-8	JBTPR-8	BTPR-8	JSD-8	TT-8	ETBPY-8
ABOXIY,	31.698,	23.086,	16.504,	11.444,	3.314,	1.015,	13.017,	27.453,	2.247,	1.756,	2.666,	11.951,	24.368

Table S2. Continuous Shape Measures' calculation for the Tb(III) ion in complex 2.

Tb structures

OP-8	1 D8h	Octagon
HPY-8	2 C7v	Heptagonal pyramid
HBPY-8	3 D6h	Hexagonal bipyramid
CU-8	4 Oh	Cube
SAPR-8	5 D4d	Square antiprism
TDD-8	6 D2d	Triangular dodecahedron
JGBF-8	7 D2d	Johnson gyrobifastigium J26
JETBPY-8	8 D3h	Johnson elongated triangular bipyramid J14
JBTPR-8	9 C2v	Biaugmented trigonal prism J50
BTPR-8	10 C2v	Biaugmented trigonal prism
JSD-8	11 D2d	Snub diphenoid J84
TT-8	12 Td	Triakis tetrahedron
ETBPY-8	13 D3h	Elongated trigonal bipyramid

Structure [ML8]	OP-8	HPY-8	HBPY-8	CU-8	SAPR-8	TDD-8	JGBF-8	JETBPY-8	JBTPR-8	BTPR-8	JSD-8	TT-8	ETBPY-8
ABOXIY,	31.788,	22.983,	16.541,	11.586,	3.073,	1.096,	13.154,	27.344,	2.040,	1.584,	2.756,	12.103,	24.236

Table S3. Continuous Shape Measures' calculation for the Tb(III) ion in complex 3.

Tb structures

OP-8	1 D8h	Octagon
HPY-8	2 C7v	Heptagonal pyramid
HBPY-8	3 D6h	Hexagonal bipyramid
CU-8	4 Oh	Cube
SAPR-8	5 D4d	Square antiprism
TDD-8	6 D2d	Triangular dodecahedron
JGBF-8	7 D2d	Johnson gyrobifastigium J26
JETBPY-8	8 D3h	Johnson elongated triangular bipyramid J14
JBTPR-8	9 C2v	Biaugmented trigonal prism J50
BTPR-8	10 C2v	Biaugmented trigonal prism
JSD-8	11 D2d	Snub diphenoid J84
TT-8	12 Td	Triakis tetrahedron
ETBPY-8	13 D3h	Elongated trigonal bipyramid

Structure [ML8]	OP-8	HPY-8	HBPY-8	CU-8	SAPR-8	TDD-8	JGBF-8	JETBPY-8	JBTPR-8	BTPR-8	JSD-8	TT-8	ETBPY-8
ABOXIY,	31.876,	22.773,	16.416,	11.604,	2.998,	1.163,	13.002,	27.432,	1.920,	1.496,	2.832,	12.121,	24.286

Table S4. Continuous Shape Measures' calculation for the Tb(III) ion in complex 4.

Tb structures

OP-8	1 D8h	Octagon
HPY-8	2 C7v	Heptagonal pyramid
HBPY-8	3 D6h	Hexagonal bipyramid
CU-8	4 Oh	Cube
SAPR-8	5 D4d	Square antiprism
TDD-8	6 D2d	Triangular dodecahedron
JGBF-8	7 D2d	Johnson gyrobifastigium J26
JETBPY-8	8 D3h	Johnson elongated triangular bipyramid J14
JBTPR-8	9 C2v	Biaugmented trigonal prism J50
BTPR-8	10 C2v	Biaugmented trigonal prism
JSD-8	11 D2d	Snub diphenoid J84
TT-8	12 Td	Triakis tetrahedron
ETBPY-8	13 D3h	Elongated trigonal bipyramid

Structure [ML8]	OP-8	HPY-8	HBPY-8	CU-8	SAPR-8	TDD-8	JGBF-8	JETBPY-8	JBTPR-8	BTPR-8	JSD-8	TT-8	ETBPY-8
ABOXIY,	32.125,	23.815,	16.621,	11.722,	3.172,	1.017,	12.868,	27.630,	2.223,	1.735,	2.626,	12.249,	24.210

Table S5. Continuous Shape Measures' calculation for the Dy(III) ion in complex 5.

Dy structures

OP-8	1 D8h	Octagon
HPY-8	2 C7v	Heptagonal pyramid
HBPY-8	3 D6h	Hexagonal bipyramid
CU-8	4 Oh	Cube
SAPR-8	5 D4d	Square antiprism
TDD-8	6 D2d	Triangular dodecahedron
JGBF-8	7 D2d	Johnson gyrobifastigium J26
JETBPY-8	8 D3h	Johnson elongated triangular bipyramid J14
JBTPR-8	9 C2v	Biaugmented trigonal prism J50
BTPR-8	10 C2v	Biaugmented trigonal prism
JSD-8	11 D2d	Snub diphenoid J84
TT-8	12 Td	Triakis tetrahedron
ETBPY-8	13 D3h	Elongated trigonal bipyramid

Structure [ML8]	OP-8	HPY-8	HBPY-8	CU-8	SAPR-8	TDD-8	JGBF-8	JETBPY-8	JBTPR-8	BTPR-8	JSD-8	TT-8	ETBPY-8
ABOXIY,	31.689,	23.108,	16.402,	11.353,	3.273,	0.975,	12.992,	27.566,	2.181,	1.735,	2.599,	11.843,	24.53

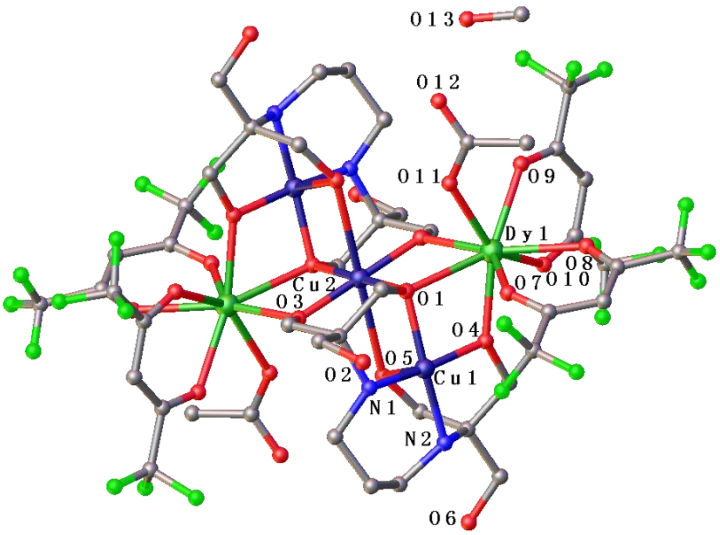

Fig. S1. Crystal structure of **5**, all H atoms are omitted for clarity.

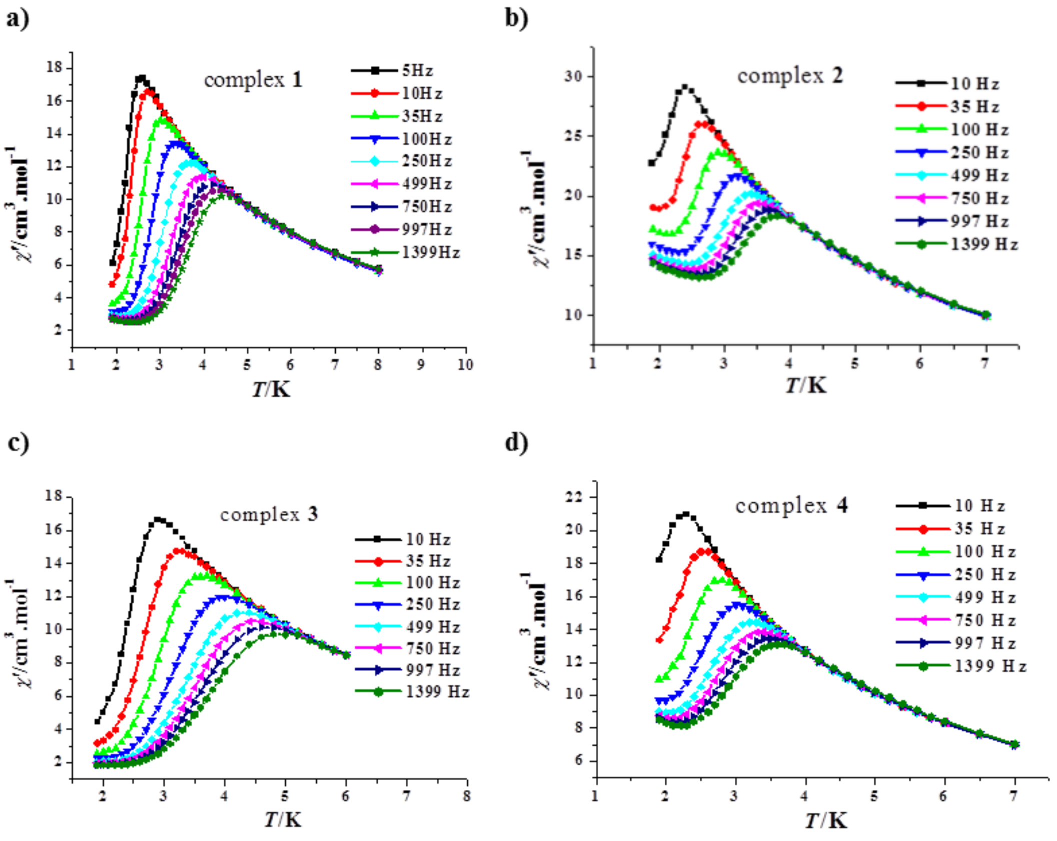

Fig. S2. Plots of χ' vs T for **1-4** (H_{dc} = 0 Oe, H_{ac} = 2.5 Oe).

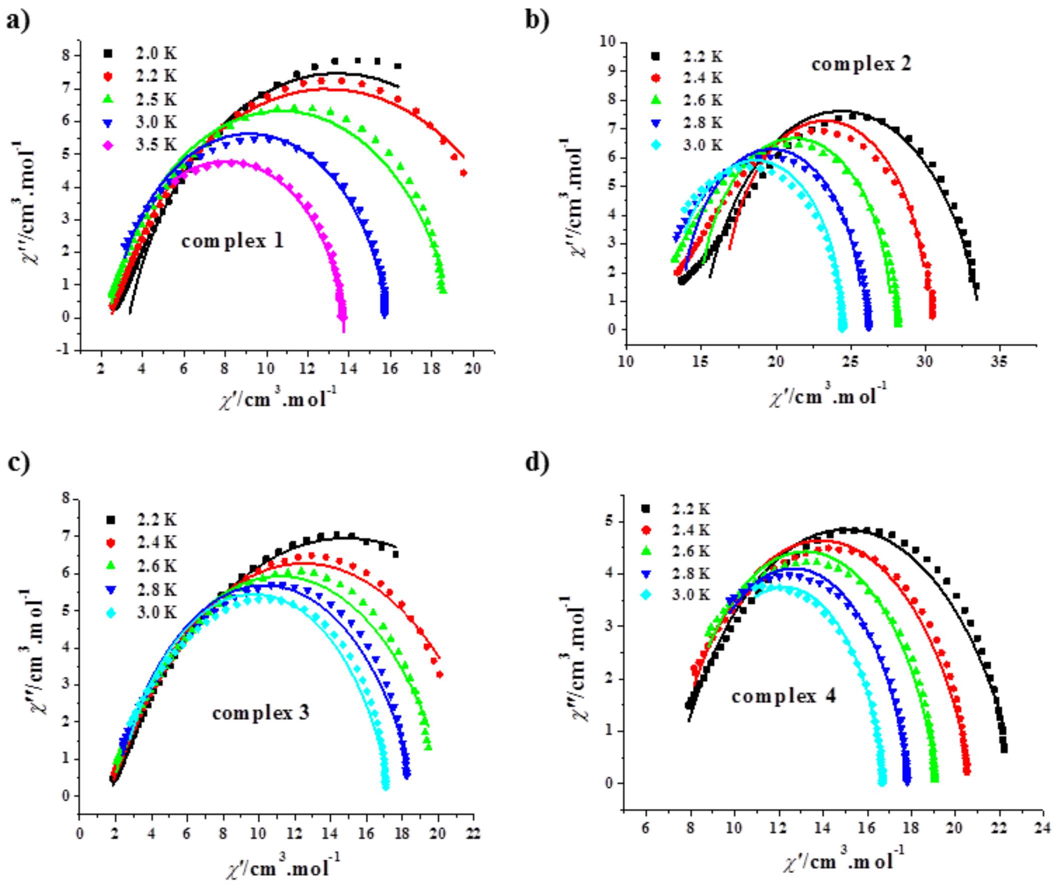

Fig. S3. Cole−Cole plots for **1-4** (H_{dc} = 0 Oe and H_{ac} = 2.5 Oe). The solid lines represent the best fitting

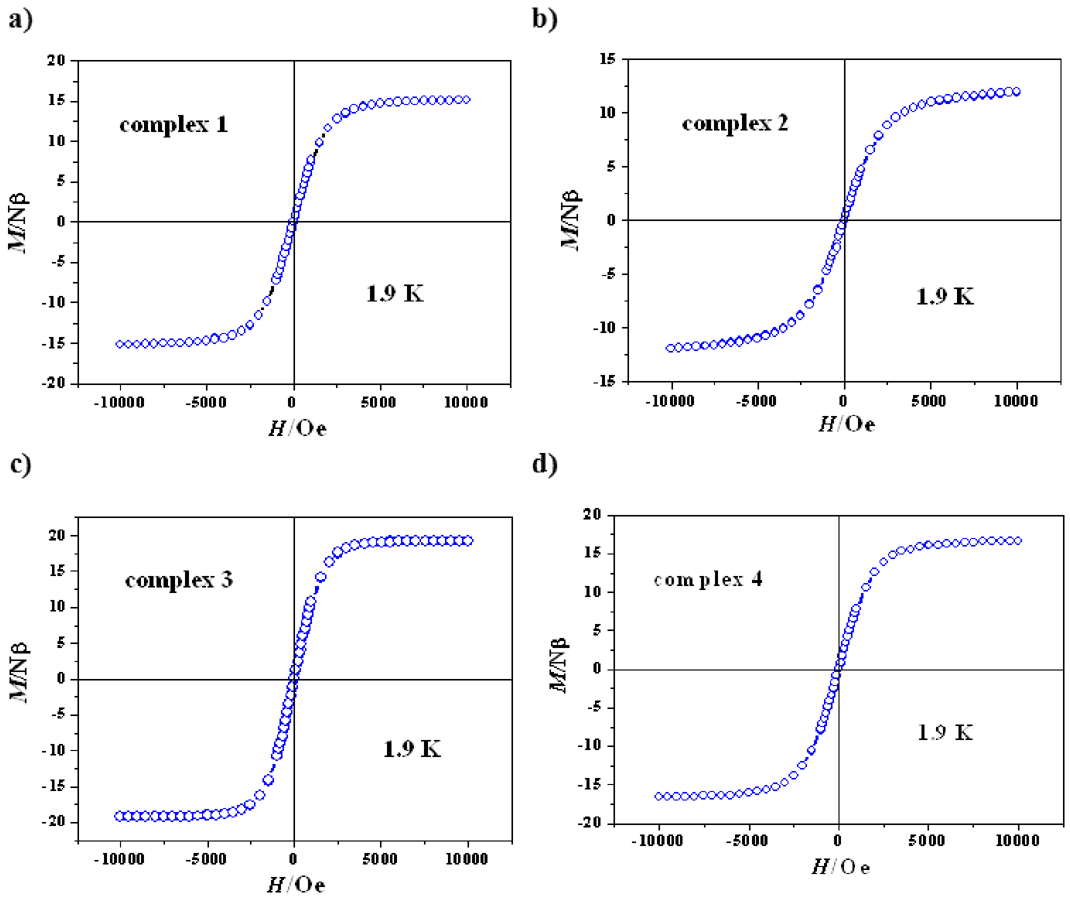

Fig. S4. Plots of *M* vs *H* at 1.9 K from −10000 to 10000 Oe for **1-4**.

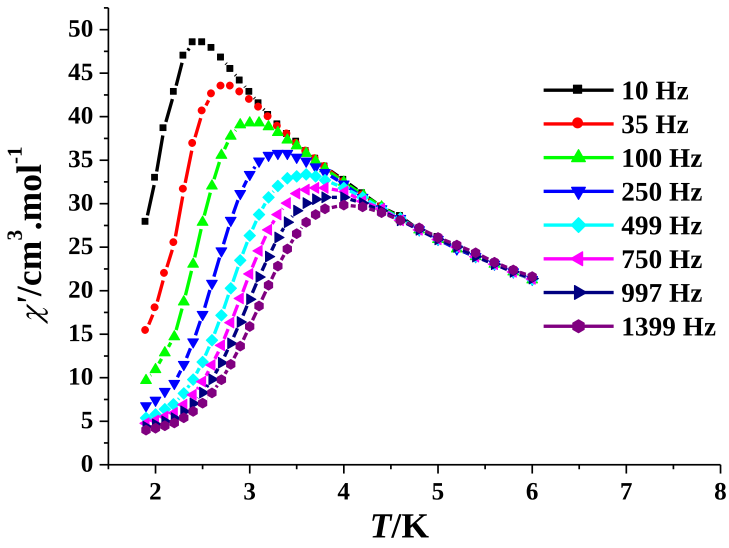

Fig. S5. Plot of χ' vs T for **5** (H_{dc} = 0 Oe, H_{ac} = 2.5 Oe).

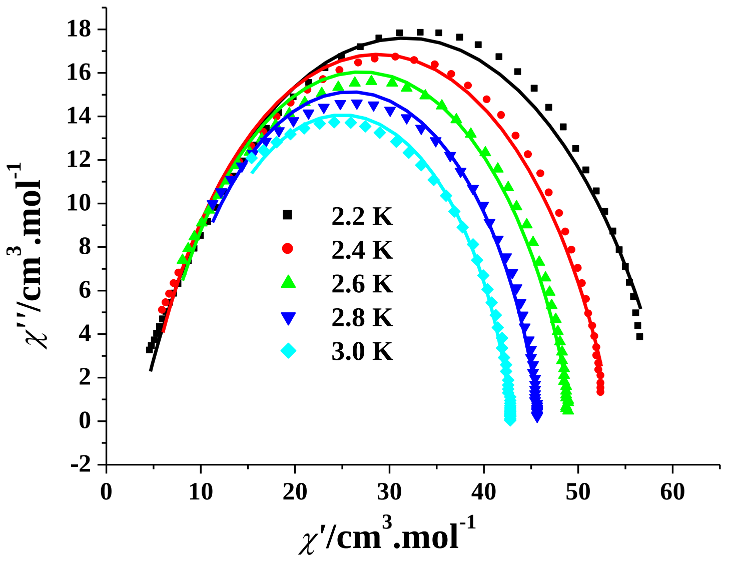

Fig. S6. Cole-Cole plot at 2.2-3.0 K for **5** (H_{dc} = 0 Oe and H_{ac} = 2.5 Oe). The solid lines represent the best fitting

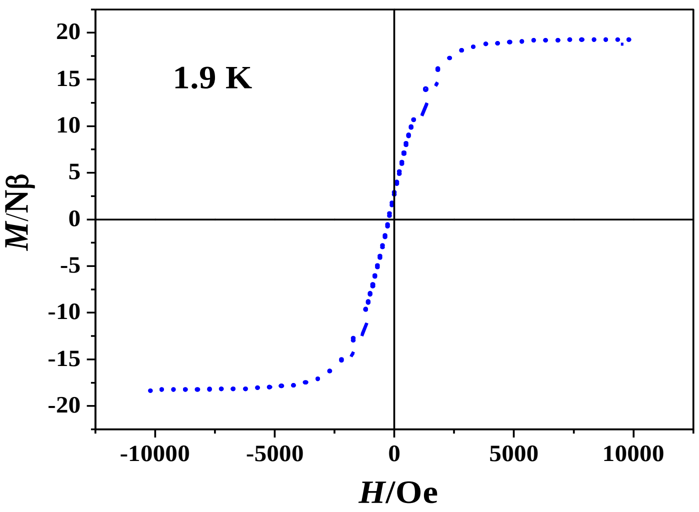

Fig. S7. Plot of *M* vs *H* at 1.9 K from −10000 to 10000 Oe for **5**.
